# An interpretable time series machine learning method for varying forecast and nowcast lengths in wastewater-based epidemiology

**DOI:** 10.1016/j.mex.2023.102382

**Published:** 2023-09-27

**Authors:** Mallory Lai, Shaun S. Wulff, Yongtao Cao, Timothy J. Robinson, Rasika Rajapaksha

**Affiliations:** a Department of Mathematics and Statistics, University of Wyoming, 1000 E University Ave, Laramie, WY, USA; b Department of Mathematical and Computer Sciences, Indiana University of Pennsylvania, 210 South Tenth Street, IN, USA; c Department of Computer Systems Engineering, University of Kelaniya, University Drive, Bulugaha Junction, Kelaniya, Colombo, Sri Lanka

**Keywords:** ARIMA model, Feature engineering, Feature selection, Gradient boosting, Pandemic management, Prediction, Stationarity, Temporal process, Time Series Machine Learning (TSML)

## Abstract

Wastewater-based epidemiology has emerged as a viable tool for monitoring disease prevalence in a population. This paper details a time series machine learning (TSML) method for predicting COVID-19 cases from wastewater and environmental variables. The TSML method utilizes a number of techniques to create an interpretable, hypothesis-driven framework for machine learning that can handle different nowcast and forecast lengths. Some of the techniques employed include:

• Feature engineering to construct interpretable features, like site-specific lead times, hypothesized to be potential predictors of COVID-19 cases.
• Feature selection to identify features with the best predictive performance for the tasks of nowcasting and forecasting.
• Prequential evaluation to prevent data leakage while evaluating the performance of the machine learning algorithm.

## Graphical abstract

**Figure:**
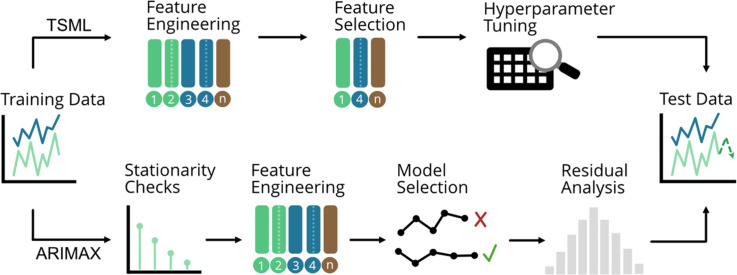

Specifications table

**Table:** 

Subject area:	*Bioinformatics*
More specific subject area:	*Wastewater-based epidemiology*
Name of your method:	*Time Series Machine Learning (TSML)*
Name and reference of original method:	*Autoregressive Integrated Moving Average with Exogeneous Variables (ARIMAX)* *Box, G.E.P., Jenkins, G.M., Reinsel,G.C.. & Ljung, G.M. (20*16*). Time series analysis: Forecasting and control (edition 5). Wiley, Hoboken, New Jersey.* *Hyndman, R.J., & Athanasopoulos, G. (2018) Forecasting: principles and practice, 2nd edition, OTexts: Melbourne, Australia. OTexts.com/fpp2. Accessed on <07-21-23>*.
Resource availability:	*N.A.*

**Method details**

## Introduction

Wastewater-based epidemiology (WBE) is a relatively quick, cost-effective, and unobtrusive tool for monitoring infections, such as Coronavirus disease 2019 (COVID-19), in a population using wastewater data. Although much of the focus has been on developing methods for accurate forecasts, creating interpretable models for epidemiologists is also important. Parametric time series models, such as auto-regressive integrated moving average with exogenous variables (ARIMAX) models, are widely considered to be interpretable. ARIMAX models have a rich history steeped in rigorous statistical theory which has made them a popular choice for forecasting. Parametric time series models, however, have drawbacks for wastewater data. First, these models require data to be collected at regular, evenly spaced intervals. In practice, wastewater data is often collected multiple times within a week. Additionally, ARIMAX models involve strict stationarity assumptions which must be met, including that the mean and variance of the response remain constant over time. ARIMAX models also rely on the use of cross-correlation functions (CCF) to determine lagged predictors. Determining predictors based upon correlation metrics makes intuitive sense, provided that the observed correlation is not spurious. Dean and Dunsmuir [1] point out that pairs of autocorrelated series, such as COVID case numbers and observed viral load in wastewater, can exhibit spurious correlations. Additionally, variable selection for ARIMAX models can be sensitive to the presence of multicollinearity [2].

Machine learning (ML) models have shown promise with forecasting and are flexible enough to handle complex and irregular data. Despite these benefits, ML models are generally criticized for lacking interpretability. Feature selection is a process where a subset of features with high performance accuracy are identified. This reduction of the feature pool can also aid in the interpretation of ML models [3]. Traditionally, few ML methods for WBE systematically incorporate temporal feature selection into model selection. Furthermore, many ML methods focus on short-term lengths, particularly one-step ahead forecasts. While these forecasts have merit, medium and long-term predictions can provide epidemiologists with enough time to prepare for potential public health challenges.

**Fig. 1:**
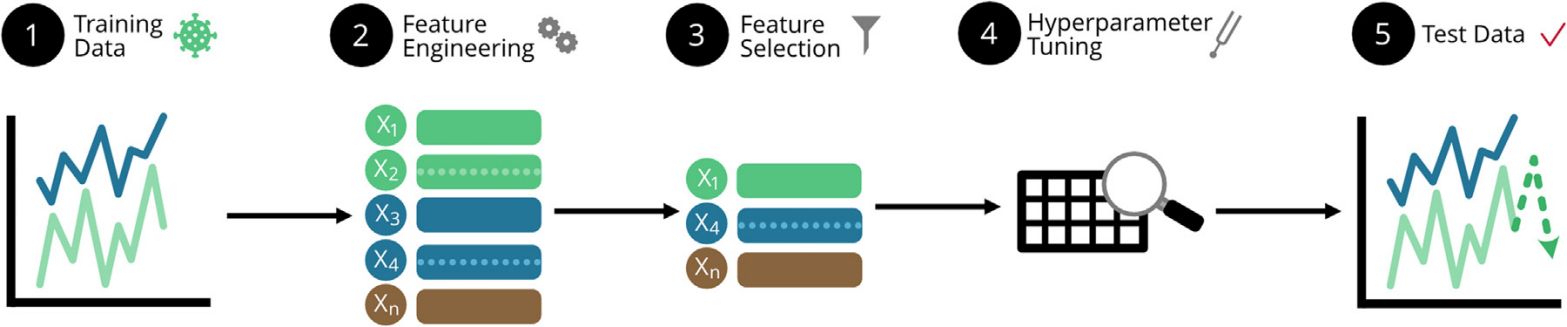
Pictorial representation of the TSML method.

The time series machine learning (TSML) method developed and illustrated in Lai et al. [4] is an interpretable, hypothesis-driven framework for machine learning that can handle varied nowcast and forecast lengths (Fig. 1). Expert knowledge is used to engineer interpretable features hypothesized to be potential predictors of COVID-19 case data, such as site-specific lead times [5]. Feature selection is then used to identify the features that have the best predictive performance for the tasks of nowcasting and forecasting. The identified features are then used to create predictions for the desired window length and task.

In Lai et al. [4], the gradient boosting machine (GBM, [6]) technique (available in the R package **gbm** [7]) is used to illustrate the feature selection framework for TSML. In tree-based methods, the predictor space is split into distinct, non-overlapping regions. To predict a new observation, the region which it belongs to is first identified, then the prediction is built from the mean or mode of the training observations in that same region [8]. Gradient boosting builds an ensemble of decision trees by sequentially building trees, correcting the performance of the previous tree [[6], [9]]. While GBM is utilized in Lai et al. [4], other ML algorithms could be substituted into the TSML approach.

**Fig. 2:**
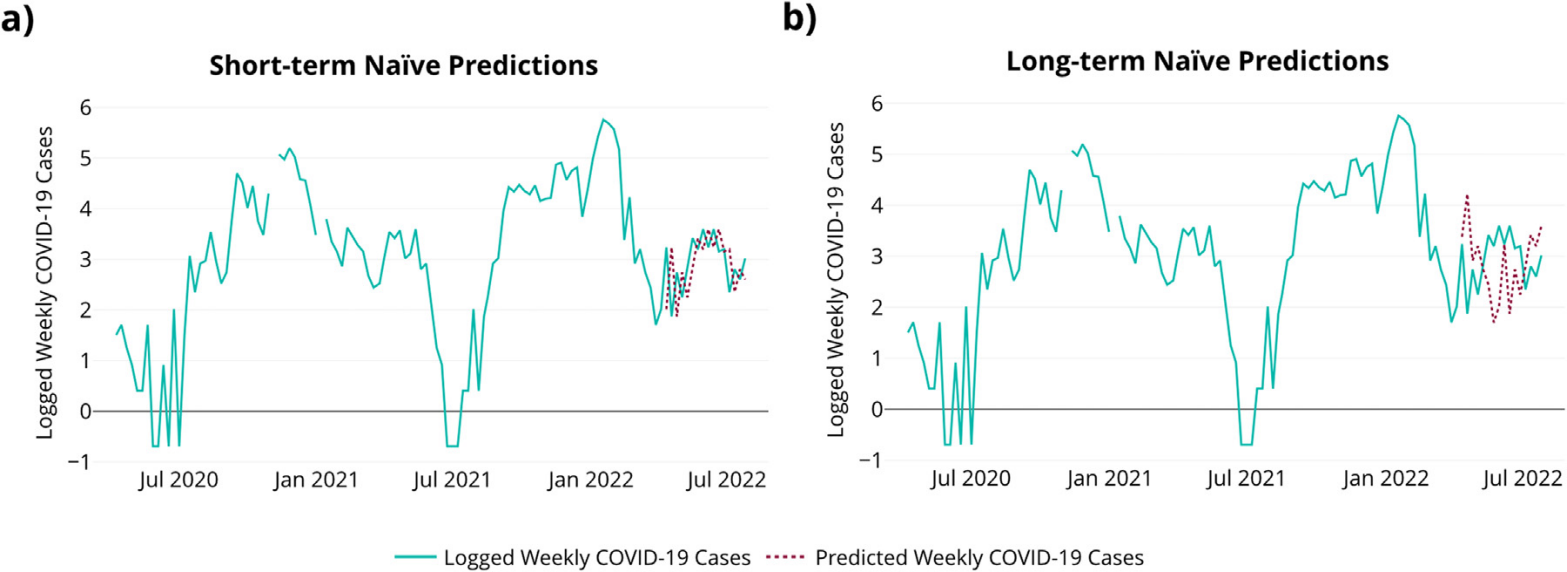
Naïve predictions for COVID-19 case data. (a) Short-term naïve predictions. (b) Long-term naïve predictions.

Lai et al. [4] contrasts the performance of TSML for predicting weekly COVID-19 cases from wastewater data using feature selection compared to manual selection of features. In this manuscript, the TSML methodology is further validated by comparing the performance to an ARIMAX model using data from Indiana, Pennsylvania. The purpose of this comparison is to characterize and differentiate the TSML method from the ARIMAX method for monitoring infections using data from WBE. The Indiana data includes cumulative weekly COVID-19 cases and the quantification of target genes indicative of the presence of SARS-CoV-2 in wastewater samples at the Indiana Borough Regional Wastewater Treatment Facility for 119 weeks. Sampling took place from April, 2020 to July, 2022. The training data consists of 96 weeks, while the testing data consists of 23 weeks. When investigating the performance of time series models, it is important to have a baseline with which to evaluate predictions. The baseline is determined from the Naïve model as shown in Fig. 2 [4]. The Naïve model uses the last available data point to form predictions. For short-term predictions, the number of COVID-19 cases at time t is used to predict the number of COVID-19 cases for the following week (t+1). For long-term predictions, the number of COVID-19 cases at time t is used to predict the number of COVID-19 cases eight weeks out (t+8).

## Time series data considerations

Analyzing and forecasting time series data requires careful consideration of the underlying temporal process. This is due to the fact that the current value of a time series variable is often influenced by its past values. Because of this, traditional ML models and validation techniques, which assume independence, are not well-suited for time series data. When developing robust time series models, it is crucial to consider the following factors.

### Stationarity

Many statistical time series methods rely on a stationarity assumption where it is assumed that the statistical properties of a temporal process, such as the mean, variance, and autocovariance, remain constant over time [10]. Violations of this assumption, due to trends or seasonality, can impact model performance and interpretability. When dealing with a non-stationary temporal process, pre-processing techniques, such as differencing and transformations, can help to satisfy the assumptions of stationarity. However, it is likely that many temporal processes will remain non-stationary despite preprocessing techniques [11]. Furthermore, while differencing is well-studied for linear models, it is unclear whether differencing remains a good strategy in the presence of nonlinearity [12].

While machine learning techniques do not necessarily rely on stationarity assumptions, many practitioners use pre-processing techniques, such as differencing, in an attempt to achieve stationarity. However, Selvam and Rajendran [13] found better performance for time series forecasting using artificial neural networks (ANN) when features were engineered to capture trend compared to removing the trend using a preprocessing step. The use of feature engineering to capture non-stationarity can improve interpretability. For instance, seasonal variables might not be specifically identified if attempts to remove stationarity from the temporal process have been undertaken. Furthermore, reversing the pre-processing steps used to create stationarity can make it difficult to interpret and visualize forecasts, particularly when double differencing is involved. For these reasons, the TSML method accommodates non-stationarity instead of trying to eliminate it. The TSML approach involves engineering features such that the features capture characteristics such as autocorrelation, seasonality, and trend.

### Forecasting versus nowcasting

**Fig. 3:**
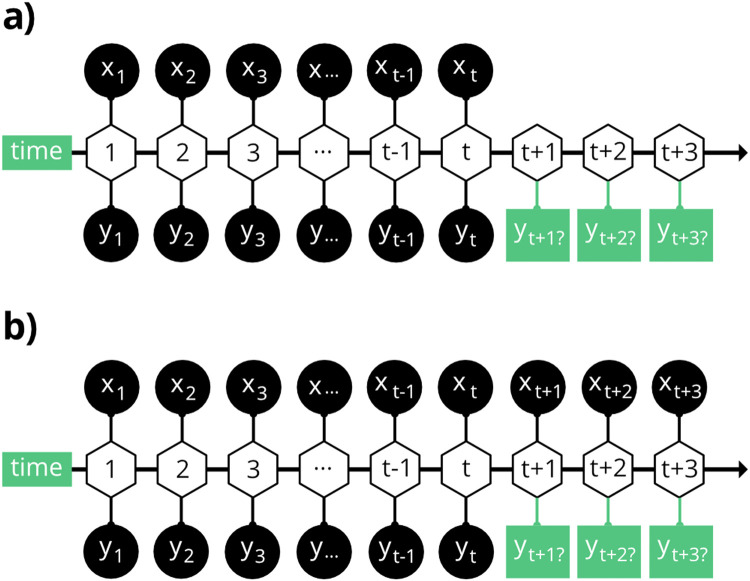
Forecasting and nowcasting COVID-19 infections with wastewater data. COVID-19 cases are represented by y_t_ and the virus load in the wastewater samples are represented by x_t_ (a) When forecasting, environmental variables are not known beyond x_t_. (b) For nowcasting, environmental variables are known for future time points. This is analogous to the situation where traditional COVID-19 testing stops, but wastewater testing continues.

Depending on the availability and accuracy of clinical COVID-19 test data, the prediction task can be classified into two types: forecasting and nowcasting (Fig. 3). With forecasting, only past and present information from both exogenous variables (ex. viral copies in wastewater data) and endogenous variables (ex. COVID-19 case data) are used to make future predictions. Forecasting is of interest to an epidemiologist who wishes to use existing information through time t to predict upcoming COVID-19 case metrics. For nowcasting, information from exogenous features is available at future time points to help predict COVID-19 cases, but it is assumed that case data in the future will not be available. Nowcasting is of interest to a public health official who would like to predict upcoming COVID-19 case metrics assuming that viral load and environmental information will continue to be available each period beyond time t, but that COVID-19 case metrics will cease to be available past some time point t, such as when testing becomes unavailable or is viewed as unreliable.

### Data leakage

Data leakage occurs when information is available during model training and validation that would not be available under ordinary prediction circumstances. Because training and validation contain more information than is given during the prediction stage, this can lead to overly optimistic estimates of model performance [14]. For time series data, this can be particularly difficult when it comes to validation techniques. If not cautious, engineered features such as lagged values of the response variable can bleed into the validation set, giving the validation set access to values of the response variable past the prediction point (Table 1).

**Table 1:** This table illustrates a toy time series consisting of values 1-10 and three engineered features, lag 1, lag 2, and lag 3. The training set consists of values above the highlighted box while the test set consists of the last three values inside the box. The values bolded in red illustrate instances of data leakage since the values 8-10 would not be known in an actual predictive scenario.

TS	Lag 1	Lag 2	Lag 3

Avoiding data leakage becomes even more complicated when developing a model that accommodates both forecasting and nowcasting. With nowcasting, exogenous variables are still available past the prediction point, whereas with forecasting, exogenous variables are not available past the prediction point. Consequently, forecasting is susceptible to data leakage of exogenous variables. However, some variables, particularly temporal variables like month, are always known past the prediction point. Overall, engineered features can be placed into three categories: (1) endogenous features that always need to be checked for data leakage, (2) exogenous features that need to be checked for data leakage when forecasting, and (3) temporal features that are always available past the prediction point. To prevent data leakage, every time the data is split for model training and validation, the features are reconstructed to ensure features do not exhibit data leakage based on the specified validation window and prediction task (Table 2).

**Table 2:** This table illustrates a toy time series consisting of values 1-10, the endogenous feature, Lag 1, the exogenous features, Predictor and Lag 1 Predictor, and the temporal feature, month. The training set consists of values above the highlighted box while the test set is inside the box. The values bolded in blue (18-20) are available during nowcasting, but unavailable during forecasting. If interest is in predictions for three months out, predictions would be made for the final line of the test set.

TS	Lag 1	Predictor	Lag 1 Predictor	month

### Temporal cross-validation

Cross-validation is a popular technique used to evaluate the performance of machine learning algorithms. Traditional k-fold cross-validation assumes observations are independent, and this assumption is obviously suspect for temporal processes. Furthermore, in traditional cross-validation, the training data is shuffled and randomly divided into multiple folds, where each fold serves as a validation set while the remaining folds are used for training. With time series data, this can lead to data leakage, where information from future time points leaks into the training set. To avoid data leakage during cross-validation, observations in the training set should precede observations in the test set.

**Fig. 4:**
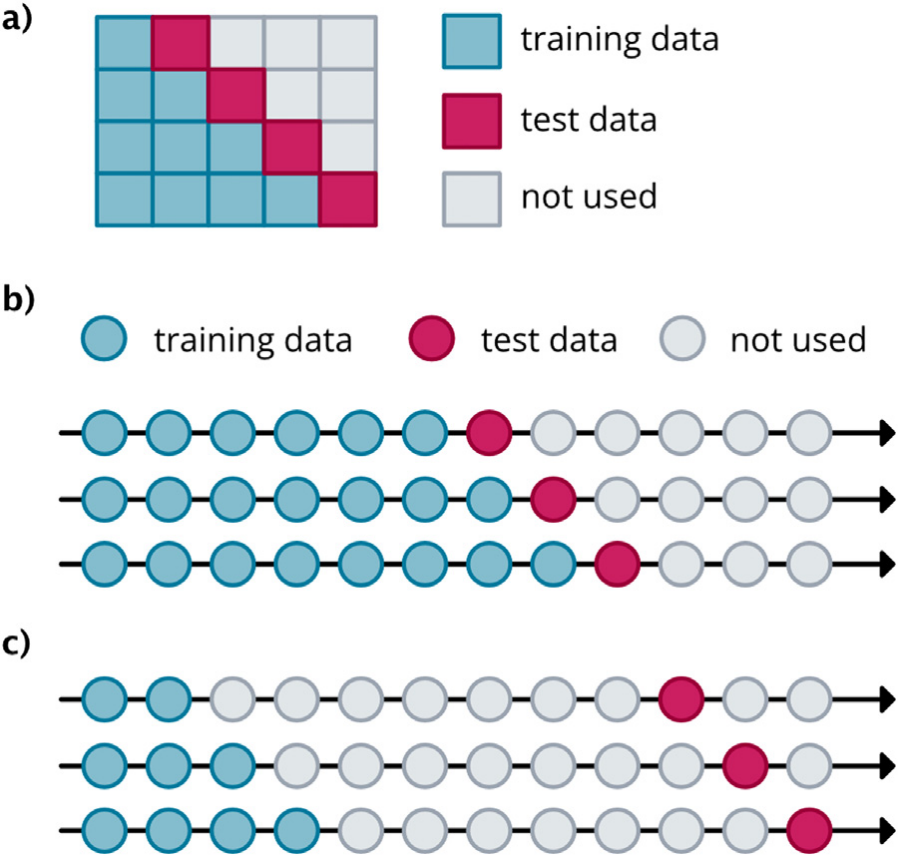
Illustration of prequential evaluation with K = 4 blocks. Adapted from Cerqueira et al. [11]. (b) Short-term rolling predictions, predicting one week in advance. (c) Long-term rolling predictions, predicting 8 weeks in advance. Adapted from Hyndman and Athanasopoulos [15].

The TSML method uses a specialized time series validation technique, prequential evaluation, that preserves the temporal order of the data for model learning and validation [11]. For the prequential method, the training data is divided into k blocks with order preserved. For the first iteration, the first block is used for training while the second block is used for validation. For each iteration thereafter, the new training set will encompass the training and validation set from the previous iteration, and the block following the new training set will become the new validation set. This continues until the last iteration where block k is used for validation (Fig. 4a). By using prequential evaluation, data leakage is avoided as the training set always precedes the test set (Fig. 4a).

## TSML method details

The steps of the TSML framework are shown in Fig. 1.  Detailed steps are as follows:

**1.** Split the data into a training and test set.
 **1.1** Set aside the test set to evaluate final model performance.
**2.** Engineer hypothesis-driven features.
 **2.1** Use expert knowledge to identify features hypothesized to be potential predictors from the relevant variables collected.
 **2.2** Consider short versus medium versus long-term features and the desired predictive task.
 **2.3** Create/engineer desired endogenous, exogenous, and temporal features.
 **2.4** Check endogenous features for data leakage.
 **2.5** If forecasting, check exogenous features for data leakage.
**3.** Use feature selection to identify those features from the candidate pool derived in Step 2 which are most useful for the desired predictive task and the given window length.

### Forward selection algorithm [16]

Let $X=\{X_{1},\;X_{2},\ldots ,X_{n}\}$ denote the feature pool, F denote the selected features, and

#(F)=s denote the number of selected features.

Initialize F=∅ to store selected features.

**Table:** 

**for** each subset size s of interest **do** // typically s is in {1,2,…,m} where m<n
**for** each feature in X**do:**
Add feature $X_{i},\;$to F.
Tune the ML algorithm with F, for the window length and predictive task of interest
// tuning involves finding optimal hyperparameters for a particular ML algorithm for feature $X_{i}$
**for** each prequential evaluation iteration **do:**
Train the model using the optimal hyperparameters and features on the training set.
Evaluate the model using RMSE on the validation set for the given window length and predictive task.
**end for**
Average the model performance measured by RMSE across prequential validation blocks.
**end for**
Add feature $X_{k}^{*}\;$to F which has the minimum RMSE and remove $X_{k}^{*}$ from X.
Store the minimum RMSE and continue if #(F)<s
**end for**

Once the above algorithm is performed for all s, plot the RMSE values by s to determine the number of features to select. Select the value $s^{*}$ where the model performance as measured by RMSE no longer improves. The selected features are then given by F where $\#\left(F\right)=s^{*}$.

**4.** Tune the final model with the selected features to identify the best hyperparameters.
 **4.1** Hyperparameter tuning algorithm

For GBM, four hyperparameters are tuned: (1) the number of trees, (2) the interaction depth, (3) shrinkage, and (4) the minimum number of observations in the terminal nodes [17]. Although there are two additional hyperparameters that can be tuned, they relate to subsampling and out-of-sample estimates. These hyperparameters should be fixed to avoid subsampling and out-of-sample estimates through GBM. Instead, subsampling should be performed manually to ensure temporally preserved ordering with a prequential evaluation process.

 **4.2** Create a hyperparameter grid consisting of relevant unique combinations of hyperparameters [18], except that that the tuning is based upon the prequential evaluation strategy.

**Table:** 

**for** each prequential evaluation iteration **do**:
**for** each row in the hyperparameter grid **do**:
Train the model using the hyperparameters specified in the current row with the selected features on the training set from Step 3.
Evaluate model using RMSE on the validation set for given window length and predictive task.
**end for**
**end for**

Average the model performance as measured by RMSE across all prequential validation blocks. Store the RMSE. Identify the hyperparameter values with the smallest mean RMSE across all prequential validation blocks.

**5.** Assess performance of the final model on the test set using the selected features and hyperparameters to forecast or nowcast for the desired horizon.

The prediction accuracy of the test set will be evaluated on a rolling origin (Hyndman, [15], Fig. 4b-c). In this scenario, the origin at which predictions are made moves forward in time, ultimately stepping through each available prediction point in the test set. As the origin moves forward one time step, the training set grows by one time step. As the training set grows, the model is refit with the hyperparameters learned from step #4 to incorporate the new observation.

A number of short and long-term features were engineered for the weekly COVID-19 cases observed in the PA data. The exogenous features included SARS-CoV-2 viral copies in wastewater, minimum ambient temperature, and temporal features, such as month and year (Table 3). It is important to note that these features were included since they were accessible. However, other features that could be of interest to the epidemiologist could easily be incorporated as well.

**Table 3:** Features engineered for TSML method.

Endogenous	Exogenous	Temporal
cases-lag1	viral copies	month
cases-lag2	Viral copies-lag1 viral copies-lag2	month-lag1
cases-lag3	viral copies-lag3	month-lag2
cases-lag8	viral copies-ma4	month-lag3
cases-lag 10	viral copies-ma8	month-lag4 year
cases-lag12	min. temperature	
cases-ma4		
cases-ma8		

**Fig. 5:**
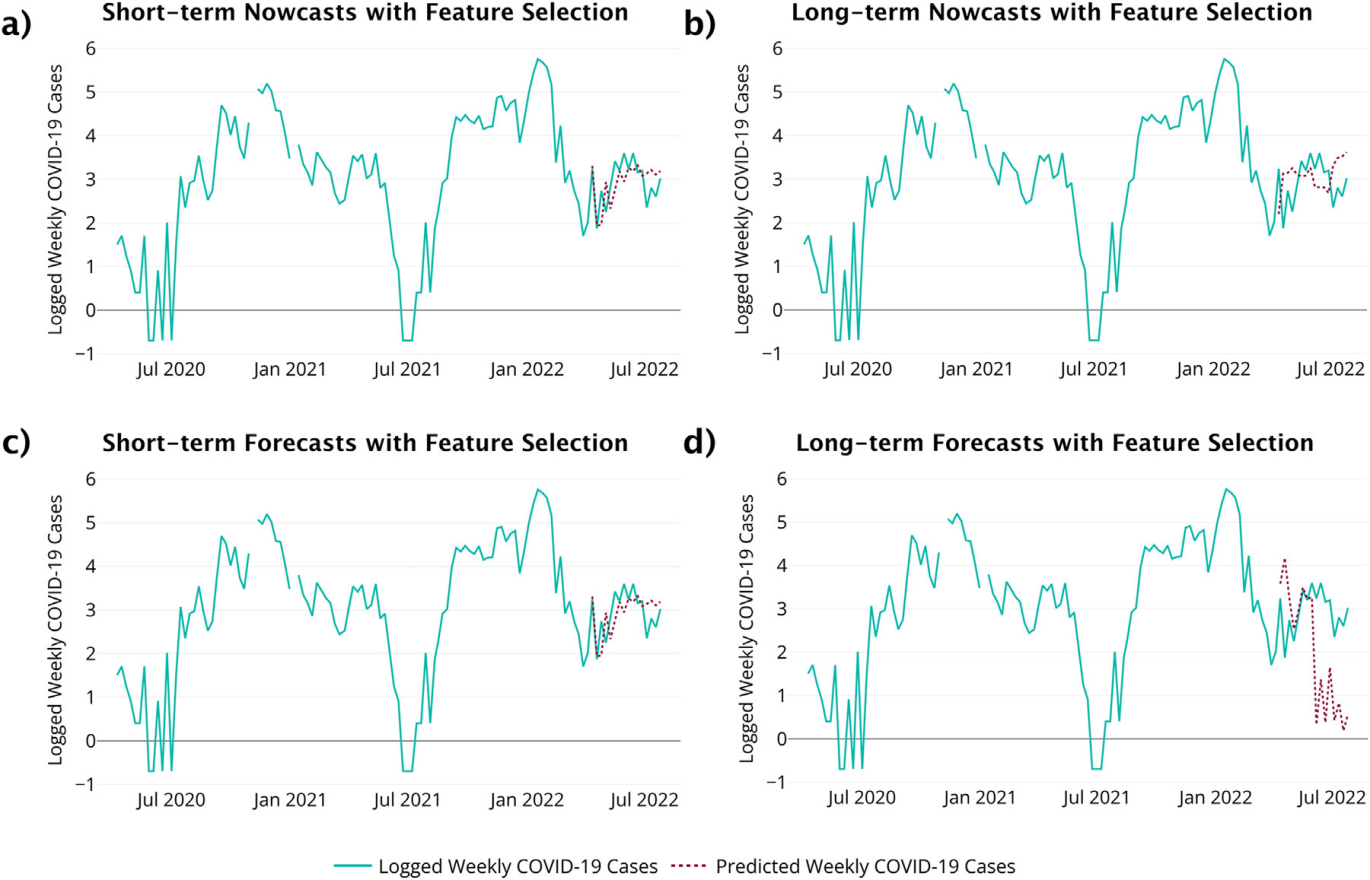
TSML method results. (a) Short-term nowcasts. (b) Long-term nowcasts. (c) Short-term forecasts. (d) Long-term forecasts.

Table 4 lists the features selected for each window length and prediction task. For short-term predictions, forward selection found the same features to be important for both forecasts and nowcasts. The number of COVID-19 cases from three weeks prior, the average viral copies from wastewater for the previous 8 weeks, the current month, and the previous week month are all important for predicting the COVID-19 cases for the next week. For long-term nowcasts, the average viral copies from wastewater for the previous 4 weeks and the month 4 weeks back were the most useful for predicting COVID-19 cases eight weeks ahead. For long-term forecasts, the current month and COVID-19 cases from eight weeks ago were the important for predicting COVID-19 cases eight weeks ahead. The performance of the selected features is illustrated in Fig. 5 with the associated performance measures in Table 6.

**Table 4:** Features selected for TSML method.

	Nowcasts	Forecasts
Short-term	Cases-lag3, month, viral copies-ma8, month-lag1	Cases-lag3, month, viral copies-ma8, month-lag1
Long-term	Viral copies-ma4, month-lag4	Month, cases-lag8

## ARIMAX method details

The ARIMAX model is a parametric model that is described by Box et al. [19] and Hyndman and Athanasopoulos [15]. However, details are provided here to illustrate the implementation of the ARIMAX model in the context of WBE and for purposes of comparison with the TSML strategy. The ARIMAX model, without seasonality, is given by [2] as

(1) $${\tilde{Y}}_{t}=\sum_{k=1}^{m}\beta_{k}{\tilde{X}}_{t,k}+E_{t},$$

${\tilde{Y}}_{t}={\left(1-B\right)}^{dy}Y_{t}$ is the differenced response of order dy,
${\tilde{X}}_{t,k}={\left(1-B\right)}^{dx,k}X_{t}^{k}$ is the differenced feature k of order dx,k,
$\beta_{k}$ is the regression coefficient for ${\tilde{X}}_{t,k}$
$\left\{E_{t}\right\}$ is an autoregressive moving average (ARMA) process satisfying $\Phi_{p}\left(B\right)E_{t}=\Theta_{q}\left(B\right)Z_{t}$,
*{*$Z_{t}\}$ is a white noise process satisfying $Z_{t}\;i.i.d\;\text{Normal}\left(0,\sigma^{2}\right)$,
$\Phi_{p}=1-\phi_{1}B-\phi_{2}B^{2}-\ldots -\phi_{p}B^{p}$ is the autoregressive operator,
$\Theta_{q}=1-\theta_{1}B-\theta_{2}B^{2}-\ldots -\theta_{q}B^{q}$ is the moving average operator,
B is the backshift operator satisfying $Bz_{t}=z_{t-1}.$

**Fig. 6:**
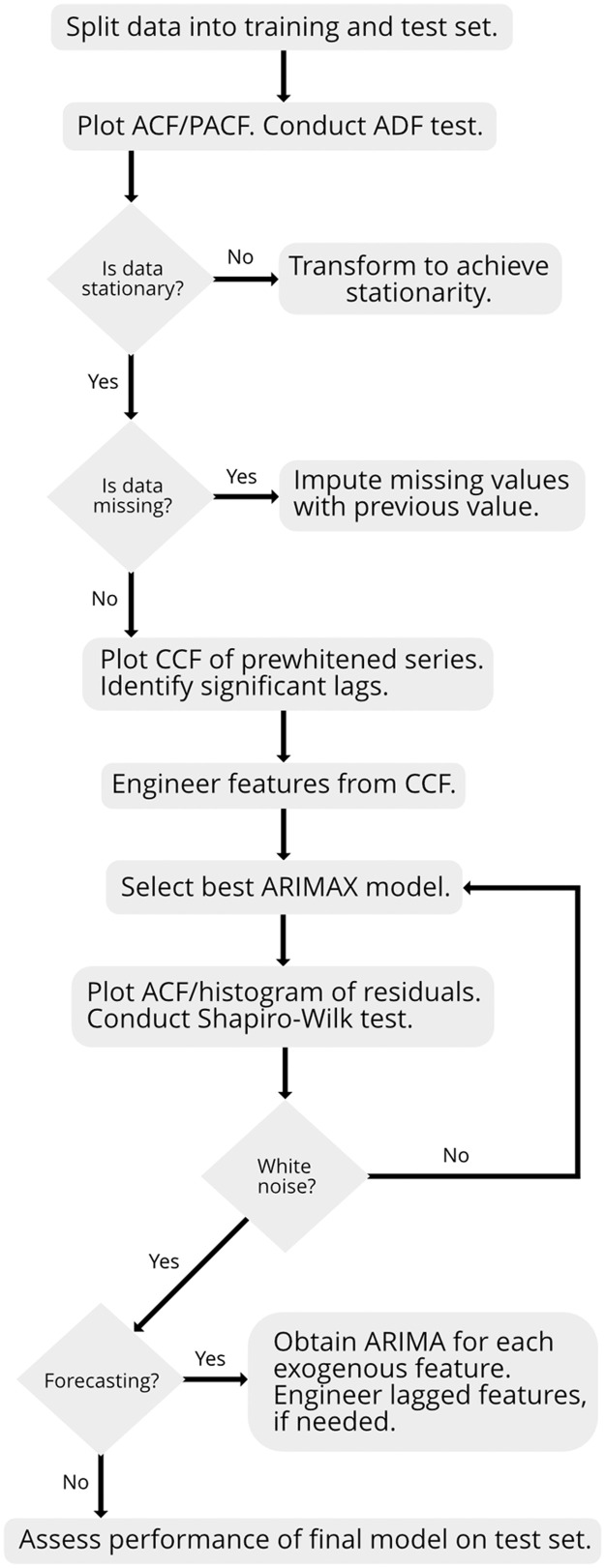
Flow diagram for the ARIMAX method.

For the ARIMAX model, the following steps were performed (Fig. 6).

**1.** Split the data into a training and test set.
 **1.1** Set aside the test set to evaluate final model performance.
**2.** Check the stationarity of the temporal process.
 **2.1** Use the autocorrelation function (ACF) and partial autocorrelation function (PACF) plots.
 **2.2** Conduct an Augmented Dickey-Fuller (ADF) Test [10].
**3.** Transform data to achieve stationarity of the temporal process, if needed.
 **3.1** Perform checks in Step 2 with transformed data.
**4.** Select exogenous features using the CCF [20].
 **4.1** If data is missing, impute by using the previous value [21].
 **4.2** Identify significant lags of features (dx,k) based on the CCF after prewhitening [10].
**5.** Engineer data-driven features.
 **5.1** Use the lags for the features identified from Step 4.
**6.** Identify the best ARMA correlation structure for the ARIMAX model errors $\left\{E_{t}\right\}$ with features identified in Step 4.
 **6.1** Use information criteria, such as corrected Akaike information criterion (AICc) [22].
**7.** Check the model assumptions of the ARIMAX model errors $\{E_{t}\}\;$using residuals.
 **7.1** Plot the ACF and histogram of residuals.
 **7.2** Conduct the Shapiro-Wilk test [23] and Ljung-Box test [24].
**8.** If forecasting, predict exogenous features, such as viral copies, beyond the training data up to the forecast horizon.
 **8.1** Obtain a separate ARIMA model for each exogenous feature.
 **8.2** Repeat Steps 2, 3, 6 to select the best ARIMA for each feature.
 **8.3** Engineer lagged features of forecasted values, if needed.
**9.** Assess performance of the final model on the test set using the selected features to forecast or nowcast for the desired horizon.
 **9.1** Evaluate the prediction accuracy of the test set using a rolling origin.

**Fig. 7:**
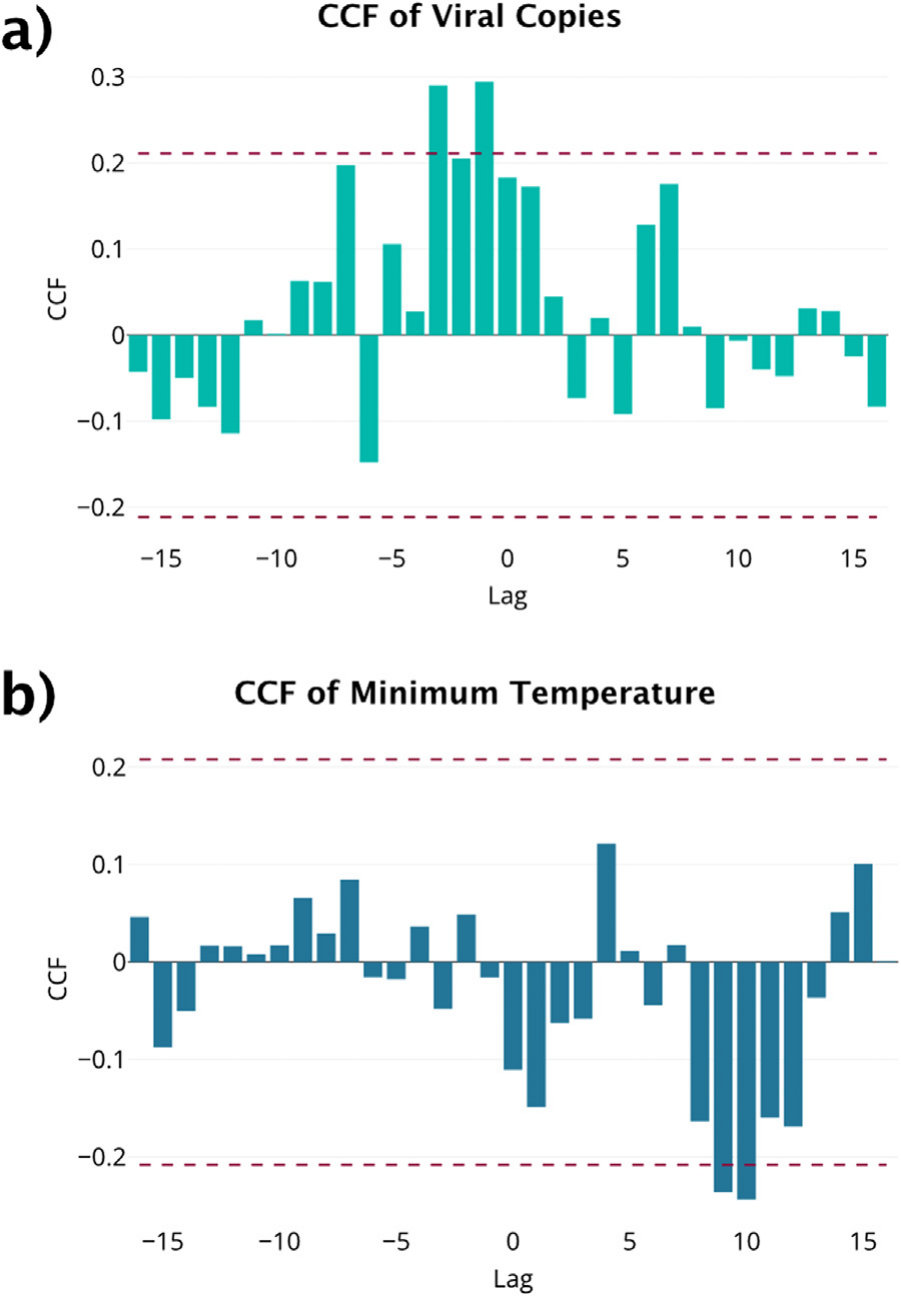
(a) CCF for prewhitened wastewater and COVID-19 case data. (b) CCF for prewhitened minimum temperature and COVID-19 case data.

Plots and stationarity tests suggest a first order difference meets stationarity assumptions for COVID-19 cases, viral copies, and minimum temperature. Since pairs of autocorrelated series can exhibit spurious correlation, prewhitening is used to separate linear relationship between two variables from the associated autocorrelations [10]. Based on the prewhitened CCF for COVID-19 cases and viral copies, lags 1 and 3 of the viral copies in wastewater are potential predictors of COVID-19 cases (Fig. 7, Table 5). No lags for minimum temperature were observed for the prewhitened CCF, so it was excluded from the ARIMAX model. It is worth noting that even if minimum temperature was included in the model, the regression coefficient for minimum temperature would not be significant or declared to be non-zero at the 0.05 level. Thus, if only significant variables were used to build the ARIMAX model, as seen in Rahman and Chowdhury [25], minimum temperature would still end up being excluded from the model.

**Table 5:** Features selected for ARIMAX model.

ARIMAX features
Viral copies
Viral copies-lag1
Viral copies-lag3

**Fig. 8:**
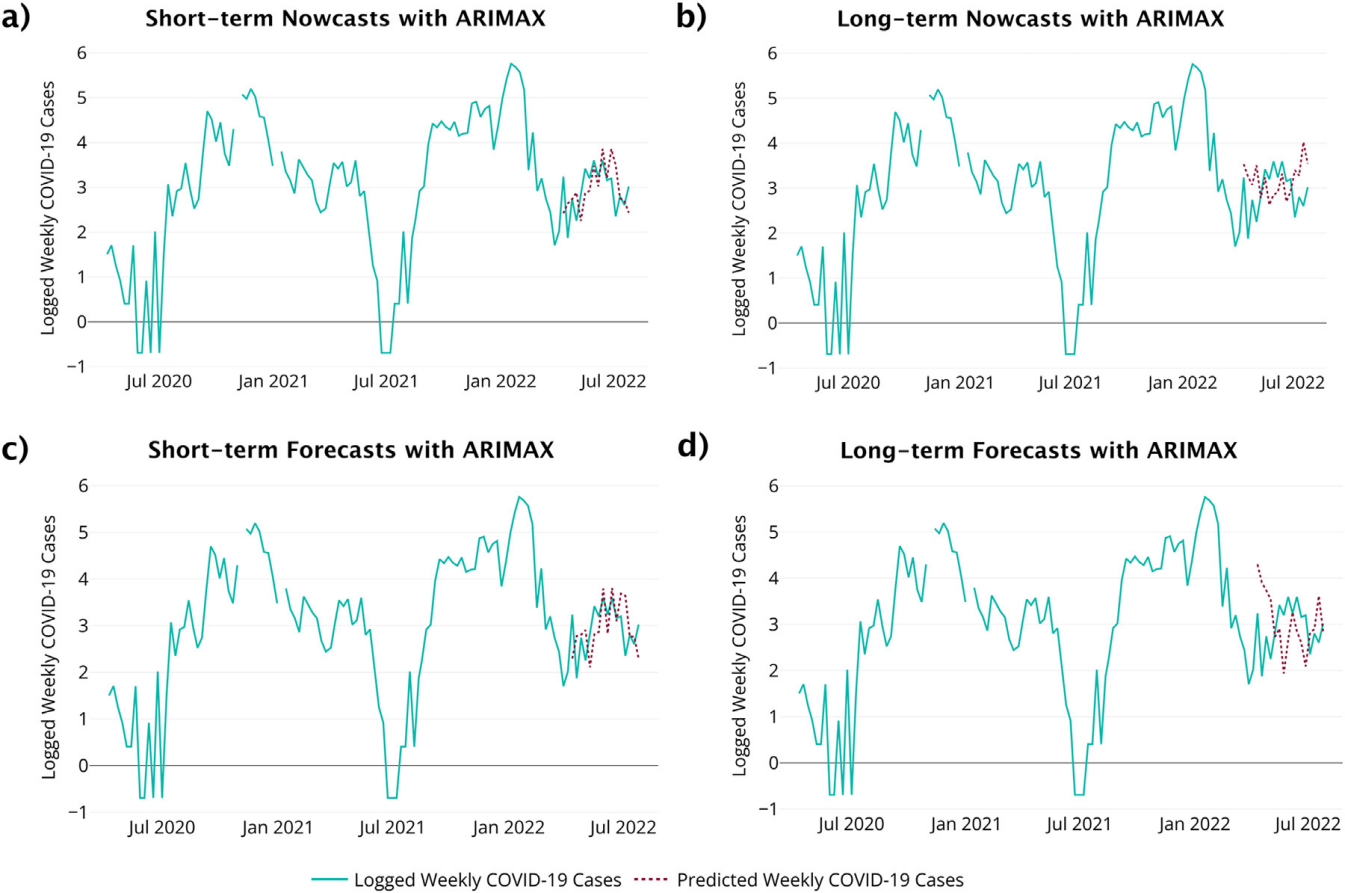
ARIMAX method results. (a) Short-term nowcasts. (b) Long-term nowcasts. (c) Short-term forecasts. d) Long-term forecasts.

The auto.arima function from the **forecast** package in R [26] found the optimal model to be an ARIMAX(0, 1, 1). The residual diagnostics from this model fit do not provide evidence of a violation of the model assumptions. For example, there is no evidence of unexplained autocorrelation in the model errors as all residuals lie within the 95% confidence limits in the ACF. The Ljung-Box test had a p-value of 0.9 and the Shapiro-Wilk test had a p-value of 0.15. The ARIMAX model performance is illustrated in Fig. 8 with its performance measures in Table 6.

**Table 6:** Performance results for wastewater data.

Nowcasts							Forecasts						
	Short-term			Long-term			Short-term				Long-term		
	RMSE	MAE	MAPE	RMSE	MAE	MAPE	RMSE	MAE		MAPE	RMSE	MAE	MAPE
Naïve	10.98	9.81	61.78	21.59	15.75	115.70	10.98	9.81		61.78	21.59	15.75	115.70
TSML	8.68	6.98	38.74	13.59	12.52	80.89	8.68	6.98		38.74	20.95	15.61	112.45
ARIMAX	10.73	8.34	50.00	16.37	13.12	92.60	11.32	9.18		57.17	22.09	17.32	118.28

### TSML method versus ARIMAX method

When comparing the features selected for the TSML method and the ARIMAX method, there are some similarities between the features. For instance, both the TSML method and the ARIMAX method found the concentration of viral copies in wastewater within the previous month to be useful predictors of COVID-19 cases. The TSML method found the average viral copies of the previous month (4 weeks) to be useful, while the ARIMAX found the average viral copies for the week prior and three weeks prior to be useful (lag 1 and lag 3). In terms of minimum temperature, neither method found it to be a useful predictor of COVID-19 cases.

Unlike the ARIMAX method, the TSML method was able to explicitly identify temporal features, lags of COVID-19 cases, and viral copies features that contain information beyond the previous month that were important for predicting COVID-19 cases. The ARIMAX method, on the other hand, did not have any features with information from more than three weeks ago, which could explain its inability to beat the Naïve model for long-term forecasts (Table 6). Although the viral copies CCF for the ARIMAX method showed a spike at lag 7, it was not deemed important and therefore was not included in the model.

Overall, the TSML features outperformed the ARIMAX features according to the performance criteria (Table 6) for this application. However, the TSML method has a major advantage over ARIMAX as it can select features based on the desired prediction task and window. ARIMAX, on the other hand, maintains the same features whether it is making nowcasts or forecasts, either short-term or long-term. This appears to hurt model performance, particularly when considering long-term forecasts. Even when ARIMAX outperforms the Naïve model, as with short-term and long-term nowcasts, the performance still lags behind that of the TSML method. While future research might be able to incorporate more refined feature selection strategies in ARIMAX, perhaps by leveraging temporal cross-validation strategies [22] or with theoretical approaches [2], the TSML method benefits from having a feature selection strategy that can be tailored to a specific prediction task and window without needing to meet specific model assumptions.

## CRediT authorship contribution statement

**Mallory Lai:** Conceptualization, Methodology, Formal analysis, Software, Visualization, Writing – original draft. **Shaun S. Wulff:** Conceptualization, Methodology, Formal analysis, Software, Writing – original draft. **Yongtao Cao:** Conceptualization, Data curation, Resources, Visualization, Writing – review & editing. **Timothy J. Robinson:** Conceptualization, Funding acquisition, Writing – review & editing. **Rasika Rajapaksha:** Methodology.

## Declaration of Competing Interest

The authors declare that they have no known competing financial interests or personal relationships that could have appeared to influence the work reported in this paper.

## Data Availability

Data will be made available on request.
